# Beneficial effects of co-treatment with dextromethorphan on prenatally methadone-exposed offspring

**DOI:** 10.1186/s12929-015-0126-2

**Published:** 2015-03-20

**Authors:** Yao-Chang Chiang, Li-Ci Ye, Kuei-Ying Hsu, Chien-Wei Liao, Tsai-Wei Hung, Wan-Jou Lo, Ing-Kang Ho, Pao-Luh Tao

**Affiliations:** Center for Drug Abuse and Addiction, China Medical University Hospital, Taichung, Taiwan; Graduate Institute of Clinical Medical Science, China Medical University, Taichung, Taiwan; Department of Pharmacology, National Defense Medical Center, Taipei, Taiwan; Center for Neuropsychiatric Research, National Health Research Institutes, Zhunan, Miaoli County Taiwan

**Keywords:** Prenatal, Opioid, Methadone, Dextromethorphan, Addiction

## Abstract

**Background:**

Heroin use among young women of reproductive age has drawn much attention around the world. Although methadone is widely used in maintenance therapy for heroin/morphine addiction, the long-term effects of prenatal exposure to methadone and preventative therapy remain unclear. For revealing this question, female pregnant Sprague–Dawley rats were sub-grouped to receive (1) vehicle, (2) methadone 5 mg/kg at embryonic day 3 (E3) and then 7 mg/kg from E4 to E20, (3) dextromethorphan (DM) 3 mg/kg, and (4) methadone + DM (the rats received methadone followed by DM treatment), subcutaneously, twice a day from E3 to E20. The body weight, natural withdrawal, pain sensitivity, ED50, conditioned place preference and water maze were conducted at different postnatal stages (P1 to P79) of offspring. The quantitative real-time RT-PCR and electrophysiology were also used to measure the gene expression of opioid receptors in the spinal cord and changes of LTP/LTD in the hippocampus, separately.

**Results:**

Prenatal exposure to methadone or DM did not affect survival rate, body weight, water maze and LTP or LTD of offspring. However, prenatal methadone significantly increased the withdrawal symptoms, pain sensitivity, addiction liability and decreased the mRNA expression of pain related opioid receptors. Co-administration of DM with methadone in the maternal rats effectively prevented these abnormalities of offspring induced by methadone.

**Conclusions:**

Our study clearly showed that co-administration of dextromethorphan with methadone in the maternal rats prevented the adverse effects induced by prenatal methadone exposure. It implies that dextromethorphan may have a potential to be used in combination with methadone for maintenance treatment in pregnant heroin-addicted women to prevent the adverse effects induced by methadone on offspring.

**Electronic supplementary material:**

The online version of this article (doi:10.1186/s12929-015-0126-2) contains supplementary material, which is available to authorized users.

## Background

Abuse of opioids causes serious social and health problems around the world. Methadone is a synthetic mu- (μ-) opioid receptor agonist that has been used for maintenance therapy in heroin addicts (including pregnant women) for more than 40 years [1]. Although methadone has been shown to be effective in reducing withdrawal symptoms and impulsive injection of opioids [2], chronic use of methadone has also exhibited addictive liability and respiratory depression in some subjects [3]. Recently, an U.S. report showed that 4.4% of pregnant women used illicit drugs in 2009–2010 [4]. This finding has drawn much attention to this population; and the effects of intrauterine drug exposure on offspring are noticed to be urgent and important issues. Children born from mothers addicted to opioids (such as heroin and morphine) have been known to suffer from higher mortality and problems with the central nervous system including dysfunction of intellectual ability or emotional control [5,6]. In addition, chronic morphine exposure during adolescence of female rats caused transgenerational effects on anxiety-like behavior and morphine-induced sensitization in the offspring [7]. Our previous studies suggest that offspring, if exposed to opioid prenatally, may experience long-term effects on behaviors and biochemical homeostasis [8]. This indicates that understanding the effects of prenatal opioid exposure on offspring and finding prevention and treatment strategies are important. Methadone is not only a synthetic μ-opioid receptor agonist but also an antagonist for *N*-methyl-D-aspartate (NMDA) receptor, with the majority of the μ-opioid receptor agonist activity residing in the R-isomer [9]. Methadone is also commonly utilized in detoxification and maintenance programs for heroin-addicted patients, including pregnant women [1,10]. Some clinical studies of methadone exposure in the pregnancy stage have found that the increase in the incidence of mortality, decrease of birth weight ranges, low head circumference, and mood, attention and cognitive deficits in the methadone-exposed infants as compared to drug-free control [11-13]. Additionally, the neonatal abstinence syndrome (NAS) is characterized to high-pitched crying, hyperactive reflexes, tremors, hypertonicity, convulsions, regurgitation, dehydration, diarrhea etc. [13]. These symptoms are like to the withdrawal syndromes in the rodents. The incidence of NAS in the newborn from MMT mother is typically 60-90%; a large of them presented severe withdrawal and need the pharmacotheraputic intervention [13]. Furthermore, the NAS increases the risk of mortality if it is left untreated [14]. The doses of methadone used for treatment of heroin addicts are wide ranges (50–100 mg/daily) [15], some cases are even closed to 200 mg/daily in previous reports and observation in our cohort [16,17]. Several studies suggested that the score of NAS, percentage of treatment for withdrawal, and duration of neonatal hospitalization were positively associated with maternal methadone dosage [18-20]. This may indicate that higher maternal methadone doses cause severe NAS. However, the issue of maternal methadone dose versus the prognosis of NAS remain unresolved, that may due to the inability to appropriately control for numerous confusing factors, such as the health status, personal neglect, poor antenatal care [13]. No matter what these findings provided, a concept that an investigation of possible strategies for reducing the dosage of methadone of maternal use or using a combination of some medicine may be helpful in decreasing adverse effects (mortality, weight gain, or NAS etc.) on offspring that were prenatally exposed to methadone.

Dextromethorphan (DM), a commonly used antitussive agent, is without opioid-like activity [21]. Both DM and its major metabolite dextrophan are NMDA receptor antagonists. DM has also been reported to treat neurologic diseases, such as non-ketotic hyperglycinemia and intractable seizure in neonates [22,23]. Results from the epidemiologic analysis of prenatal exposure of therapeutic dose of dextromethorphan (117.2 mg, the maximum dose is 120 mg/daily with 30 mg for 4 times [15]) showed no evidence of teratogenicity in infants [24]. Studies have shown that DM reduced morphine reward as measured by CPP test, and this might be related to DM’s antagonizing effect on NMDA receptors that are involved in the activation of mesocorticolimbic dopaminergic systems [25,26]. Our previous studies also demonstrated that co-administration of DM with morphine during pregnancy and throughout lactation reduced several morphine-induced adverse effects [27,28] and could prevent higher vulnerability to inflammatory thermal hyperalgesia in rat offspring [29].

Although methadone is also a μ-opioid agonist like morphine, the effects of DM in prenatally methadone-exposed offspring have not been explored. Therefore, in the present study, we aimed to investigate if the prenatal co-administration of DM with methadone prevents or decreases the possible adverse effects induced by methadone on the offspring, such as withdrawal symptoms, changes of pain sensitivity, tolerance, addiction liability (reward), memory impairment, etc.

## Methods

### Animals

Pregnant Sprague–Dawley rats (BioLASCO Taiwan Co., Taipei, Taiwan) and their offspring were used in this study. All animals were housed in an animal room with a 12-h light/dark cycle (light on 07:00–19:00), a temperature of 25°C, and humidity of 50 ± 10%. Pregnant rats were kept individually in separate cages, and their male offspring were housed 2–3 per cage after weaning. A diet (Prolab 2500 Rodent 5P14, LabDiet, PMI Nutrition International, St. Louis, MO, USA) and water were provided *ad libitum.* The care of animals was carried out in accordance with institutional and international standards (Principles of Laboratory Animal Care, NIH); the animal protocol was approved by the IACUC of the National Health Research Institutes (NHRI-IACUC-099087-A). All experiments were performed following the schedule below.

### Drugs

Methadone HCl (USP, Maryland, MD, USA) or dextromethorphan HBr (Sigma Aldrich, St. Louis, MO, USA) were dissolved in sterilized distilled water and were administered subcutaneously (*s.c.*) in a volume of 1.0 ml/kg of body weight.

### Prenatal treatment

Pregnant Sprague–Dawley rats, weighting 200–250 g (10–12 weeks old), were assigned to four groups randomly. Each group was *s.c.* injected with either vehicle or drug(s) during the gestational period (E3–E20). Vehicle control group rats received distilled water, 1 ml/kg (*s.c.*), twice a day. Methadone-group rats received 5 mg/kg at E3 and then 7 mg/kg from E4 to E20 (*s.c*), twice a day. DM group rats received 3 mg/kg of DM (*s.c.*), twice a day. In the methadone + DM group, the rats received methadone followed by DM treatment twice a day. The offspring were weaned at day 28 after birth and housed 2–3 per cage after weaning until use. The offspring from the same dam were randomly assigned to different experiments to avoid the litter effect (the female offspring were not used in this study).

### Determination of natural withdrawal behaviors

Natural withdrawal behaviors of the offspring were observed for 15 min within 24 h after birth. One pup was chosen randomly and removed from the dam before being placed on a white filter paper. Head moves, moving paws, rolling, stretching, twisting, and walking behaviors were observed and counted for 15 min. There were at least 11 pups in each group.

### Determination of pain sensitivity of offspring on p30 and p60

Tail-flick test: A light beam was focused on an animal’s tail until that the rat flicked its tail. The recorded time was a measure of the pain threshold. The tail-flick apparatus (Columbus Instrument, Columbus, OH, USA) was used in the study. The basal latency of most rats was around 3–3.5 sec; a cutoff time of 10 sec was used to avoid tissue damage.

Hot-plate test: In this study, a hot-plate apparatus (IITC Life Science Inc., Woodland Hills, CA, USA) was used. The animal was placed on the hot plate and the time was measured until the animal started to shake or lick its hind paw or jump from the hot plate. To avoid tissue damage, the animal was removed immediately from the metal surface after it showed pain-related behavior or reached the cutoff time (30 sec).

### Determination of ED_50_ and degree of tolerance after sub-chronic treatment of’ methadone in adult offspring

The ED_50_ value was used as an index of the antinociceptive effect of methadone and this was determined by the up-down method [30] with the tail-flick method. Details were shown in the Additional file 1. The degree of tolerance was defined as the ratio of ED_50_ after sub-chronic treatment of methadone (4 mg/kg, *s.c.*, b.i.d. for 6 days) (day 79) and ED_50_ before CPP experiment and sub-chronic methadone treatment (day 61).

### Determination of the rewarding effect of methadone in adult offspring by conditioned place preference (CPP) test

In this study, we used the CPP test to evaluate methadone-induced reward in the adult male offspring (p64-p71). Details are shown in the Additional file 1. For CPP conditioning, each rat was given saline in the morning and put in one chamber of the CPP box for 40 min and was given drug (methadone, 4 mg/kg, *s.c.*) in the afternoon and put in another chamber of the CPP box for 40 min everyday for 6 days. CPP tests were performed and recorded for 15 min on the day before and the day after conditioning for 6 days.

### Water maze test

The water maze was circular, had a diameter of 180 cm and a depth of 60 cm, and was filled with water (21–23°C) to a depth of 40 cm. Spatial cues were located on the walls. The total pool was divided into 4 quadrants (NE, SE, SW, NW), which were designated as zones 1–4, respectively. The transparent platform (2 cm below the surface of the water) was placed in the zone 1 area (NE quadrant). Rats received 5 days of training for two trials per day. The time for the animal to find the platform was recorded, and the maximal duration for each trial was 60 sec; then the animal was put on the platform for 10 sec to allow the animal to look around. On day 6, the platform was removed and the time the animal spent in zone 1, the previous location of the platform, was determined. All parameters were automatically recorded and analyzed by video tracking software (Etho vision, Noldus, Leesburg, VA, USA).

### Hippocampal LTP and LTD determination by electrophysiology

The details of the electrophysiology recording protocol are described in the Additional file 1. Briefly, transverse 350-μm-thick hippocampal slices were prepared from p14-p21 male offspring using a commercial vibratome (DTK-1000, Dosaka, Kyoto, Japan). Tungsten bipolar electrodes (FHC, Bowdoin, ME, USA) were placed in the stratum radiatum of the hippocampal CA1 region to stimulate the Schaffer collateral pathway directly. Field excitatory postsynaptic potentials (fEPSPs) were recorded and analyzed offline. After 30 min of stable baseline recording, a high-frequency stimulation (HFS, consisting of two trains of 100 Hz bursts, 1 s in duration and with a 1 min span between the two trains) or a low-frequency stimulation (LFS, 1 Hz for 15 min) was applied to Schaffer collaterals to induce long-term potentiation (LTP) or long-term depression (LTD) of EPSPs, respectively.

### Determination of expressions of mRNAs by quantitative real time RT-PCR (qPCR)

Details of qPCR protocol are described in the Additional file 1. Briefly, total RNA was extracted from spinal cord tissue samples using TRIzol reagent (Invitrogen, Carlsbad, CA, USA). First-strand cDNA was synthesized from 1 μg of the total RNA with an iScript^TM^ cDNA Synthesis Kit (Bio-Rad Laboratories, Hercules, CA, USA). The SYBR green system (SsoFast^TM^ EvaGreen, Bio-Rad) was used to determine the Ct (threshold) value. Primer sequences for NOP receptor (*Oprl1*) (designed by Beacon Designer 7.6 [PREMIER Biosoft, Palo Alto, CA, USA] with NCBI reference sequence NM_031569.3), μ-opioid receptor (*Oprm1*) and glyceraldehyde-3-phosphate dehydrogenase (*Gapdh*) (housekeeping gene) are presented in Table 1. A three-step run protocol was used: (i) 1 sec at 98°C; (ii) quantification program repeated 40 times (1 sec at 98°C; 10 sec at 57°C); (iii) melting curve program (70–93°C with a heating rate of 0.5°C per 10 sec). The Ct value of each gene was normalized with their *Gapdh.* ΔΔCt = ΔCt (target gene_treat_- GAPDH_treat_)-ΔCt (Control_control_-GAPDH_control_). The fold change was measured as 2^−ΔΔCt^.

**Table 1:** **Primers for** ***Oprl1*** **,** ***Oprm1*** **and** ***Gapdh*** **for qPCR**

**Gene**	**Primer**	**Sequence**	**Position**	**Product (bp)**	**Reference**
*Oprl1*	Forward	CTGGGAGGTCTTGTATGG	268–360	93	Designed by software
	Reverse	CTGTGACTAGCATTGAGGA			
*Oprm1*	Forward	GTAGTGGGCCTCTTCGGAAAC	447–521	75	[59]
	Reverse	GTTGGTGGCAGTCTTCATTTG			
*Gapdh*	Forward	AACGACCCCTTCATTGAC	169–359	191	[60]
	Reverse	TCCACGACATACTCAGCAC			

### Statistical analysis

Data are expressed as mean ± SEM. Student *t*-test, one-way or two-way ANOVA, repeated measures one-way ANOVA, and the Newman-Keuls test or Bonferroni post-tests were used to analyze the data. A difference was considered to be significant if p < 0.05.

## Results

### Prenatal exposure to methadone or DM did not influence the survival rate or body weight of offspring

**Figure 1:**
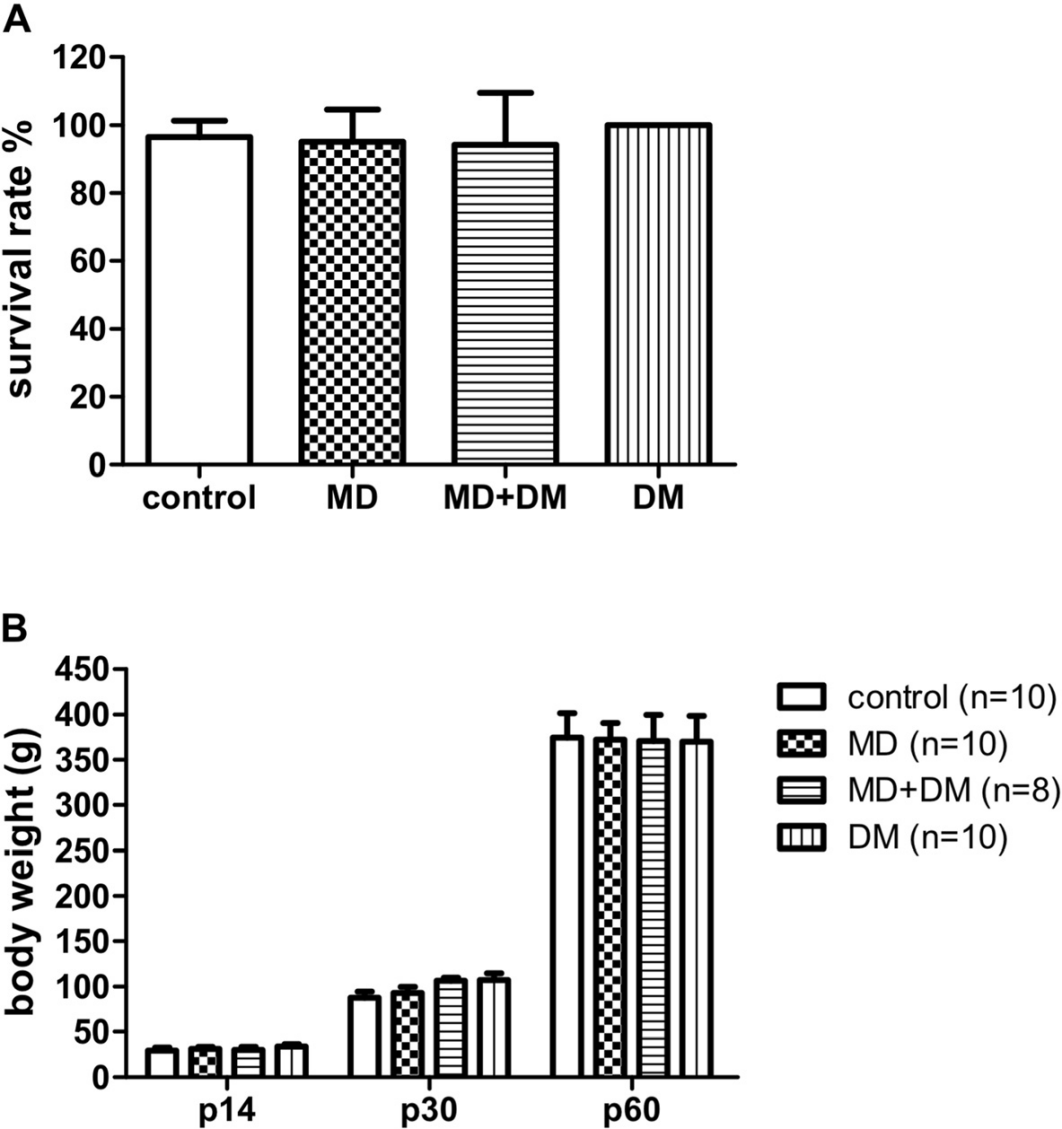
**Prenatal exposure to methadone did not affect the survival rate and body weight. (A)** The survival rate of offspring on day 7 after birth or **(B)** the body weight at different ages of male offspring. Data are presented as mean ± S.E.M. (n ≥ 8).

As shown in Figure 1A, the survival rate of offspring on day 7 after birth for the methadone, DM, and methadone + DM groups did not differ significantly from the control group (F_(3,27)_ = 3.19; p = 0.08). The litter sizes in all tested groups were not significantly different in current study. Similarly, the body weights of all tested groups at p14 (F_(3,28)_ = 0.47; p = 0.71), p30 (F_(3,34)_ = 2.30; p = 0.09), and p60 (F_(3,34)_ = 0.006; p = 0.999) showed no significant difference (Figure 1B). These results indicate that prenatal exposure to methadone, DM, or methadone + DM at the doses in the current study did not change the survival or growth rate of offspring.

### Prenatal co-administration of DM with methadone prevented the increase of natural withdrawal behaviors of offspring within 24 h after birth

Natural withdrawal behaviors of the offspring were shown on Table 2. We found that the total counts of natural withdrawal symptoms were significantly higher in the methadone group (71.8 ± 17.8) than these in the control group (17.3 ± 4.0). Co-administration of DM with methadone reduced these natural withdrawal symptoms to 38.3 ± 4.2, which was not significantly different from that of the control group. Surprisingly, prenatal exposure to DM alone also increased the natural withdrawal symptoms in offspring to 60.5 ± 14.0, as shown on Table 2.

**Table 2:** **Natural withdrawal behaviors of offspring within 24 h after birth**

	**Control**	**Methadone**	**Methadone + DM**	**DM**
**Natural withdrawal symptoms**		**Count (Mean ± SEM)**		
Head moves	10.6 ± 2.5	22.8 ± 6.7	23.3 ± 2.9	44.3 ± 11.1**
Paws moving	1.7 ± 0.2	44.8 ± 12.5***	8.2 ± 2.6^##^	14.1 ± 4.0^##^
Stretching	0.0 ± 0.0	0.0 ± 0.0	0.0 ± 0.0	0.17 ± 0.17
Twisting	4.6 ± 1.7	3.2 ± 1.1	6.8 ± 1.4	0.8 ± 0.5
Rolling	0.4 ± 0.4	0.8 ± 0.5	0.0 ± 0.0	1.3 ± 1.0
Walking	0.0 ± 0.0	0.17 ± 0.17	0.0 ± 0.0	0.0 ± 0.0
Total score	17.3 ± 4.0	71.8 ± 17.8*	38.3 ± 4.2	60.5 ± 14.0*

Natural withdrawal behaviors of the offspring were observed and counted for 15 min within 24 h after birth. Data are presented as mean ± S.E.M. (n ≥ 11). One-way ANOVA and Newman-Keuls test were used to analyze the data. *P < 0.05, **p < 0.01, and ***p < 0.001, compared to the control group; ^##^p < 0.01, compared to the methadone group.

### Prenatal methadone exposure increased the pain sensitivity of offspring, and co-administration of DM with methadone prevented this effect

**Figure 2:**
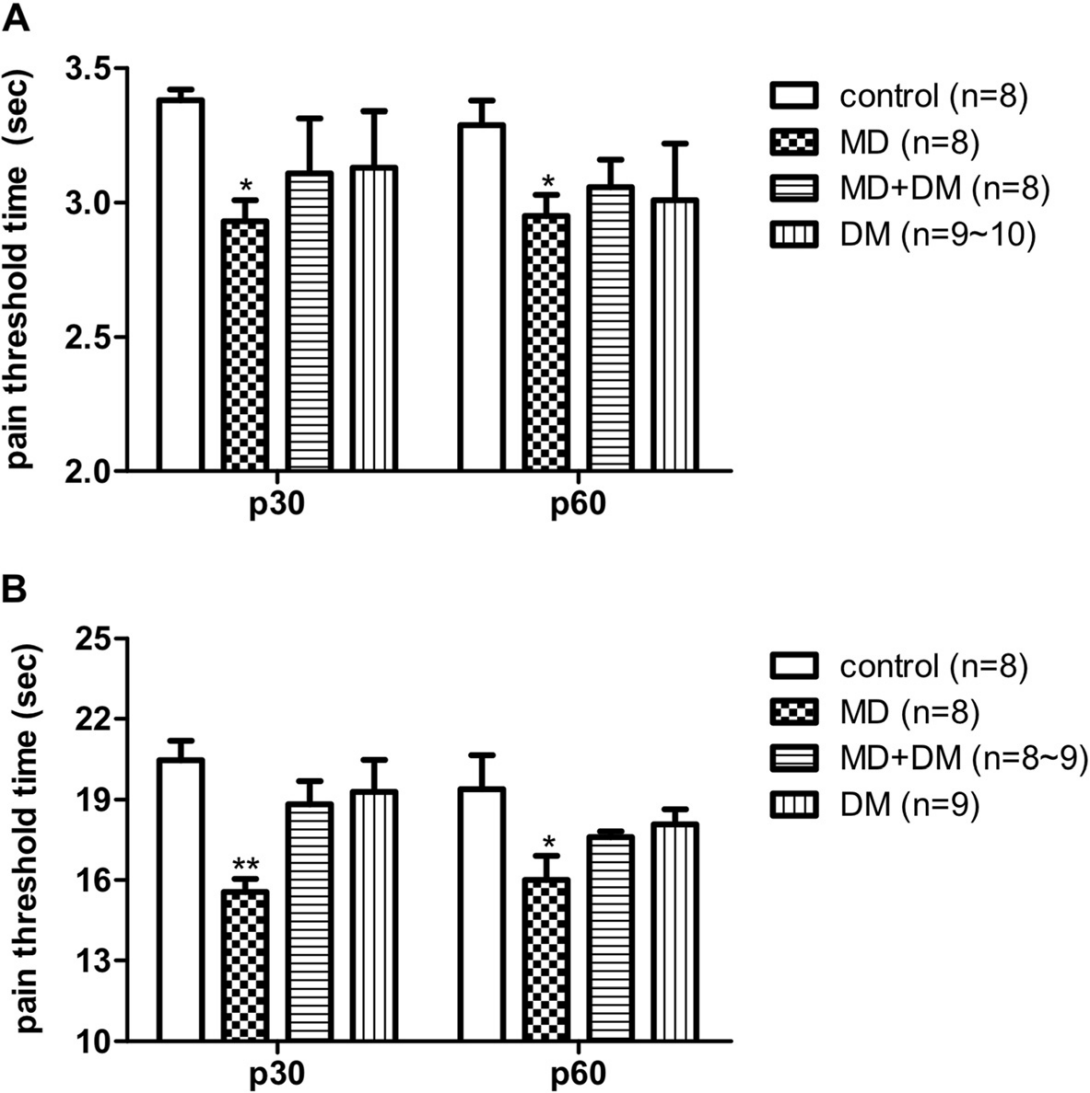
**The effects of pain threshold in prenatal drug exposure male offspring.** Prenatal exposure to methadone (MD) decreased the pain threshold at different ages (p30 or p60) in male offspring and prenatal co-administration of methadone and dextromethorphan (MD + DM) prevented this effect. Acute heat nociceptive responses were determined by **(A)** tail-flick test and **(B)** hot-plate test. Data are presented as mean ± S.E.M. (n ≥ 8). *P < 0.05 and **p < 0.01 when compared to the control group.

Prenatal methadone-exposure significantly decreased the pain threshold of offspring determined by either tail-flick (Figure 2A) or hot-plate test (Figure 2B) on p30 (F_(3,31)_ = 5.00; p < 0.05) or p60 (F_(3,29)_ = 2.85; p < 0.05). Prenatal exposure to DM did not alter the pain threshold of offspring significantly. However, prenatal exposure of DM in conjunction with methadone from E3-E20, prevented the changes induced by prenatal methadone exposure alone either partially or completely in the tail flick and hot plate tests, respectively.

### The mRNA levels of pain-related receptors in the spinal cord of male offspring

**Figure 3:**
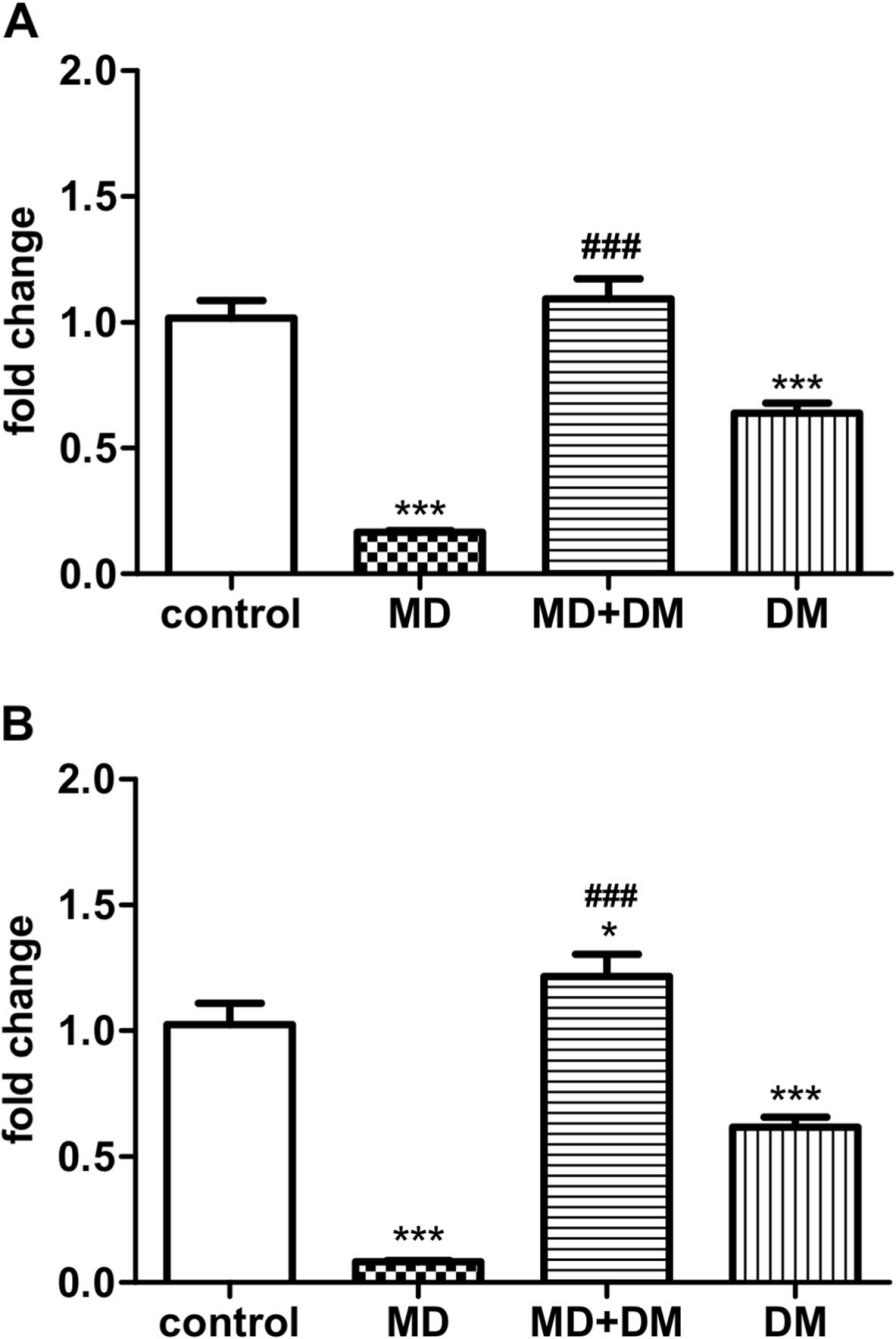
**The mRNA expression of pain-related opioid receptors in the spinal cord of offspring.** The mRNA level of **(A)** mu-opioid receptor (MOR); and **(B)** nociceptin receptor (NOPR); in the spinal cord of prenatal drug(s) exposed male offspring at P30. Data are presented as mean ± S.E.M. (n ≥ 8). *P < 0.05, **p < 0.01 and ***P < 0.001 when compared to the control group; ###p < 0.001 when compared to the methadone (MD) group.

In order to reveal the possible mechanisms by which prenatal methadone affects pain sensitivity, we investigated the gene expression of μ-opioid receptors (MOR) and nociceptin/orphanin FQ receptors (NOPR) in the spinal cord of male offspring. Our results showed that prenatal exposure to methadone caused a significant decrease in the gene expression (mRNA level) of MOR (F_(3,28)_ = 56.35; p < 0.001) and NOPR (F_(3,28)_ = 60.53; p < 0.001) (Figure 3A and B). In the results of prenatal exposure to DM alone, there were also slight but significant decreases in the mRNA levels of MOR and NOPR. However, co-administration of DM with methadone in the maternal rats significantly prevented the decrease in mRNA levels of these two pain-related receptors in the spinal cord of offspring induced by prenatal methadone exposure.

### Rewarding effects of methadone in male adult offspring

**Figure 4:**
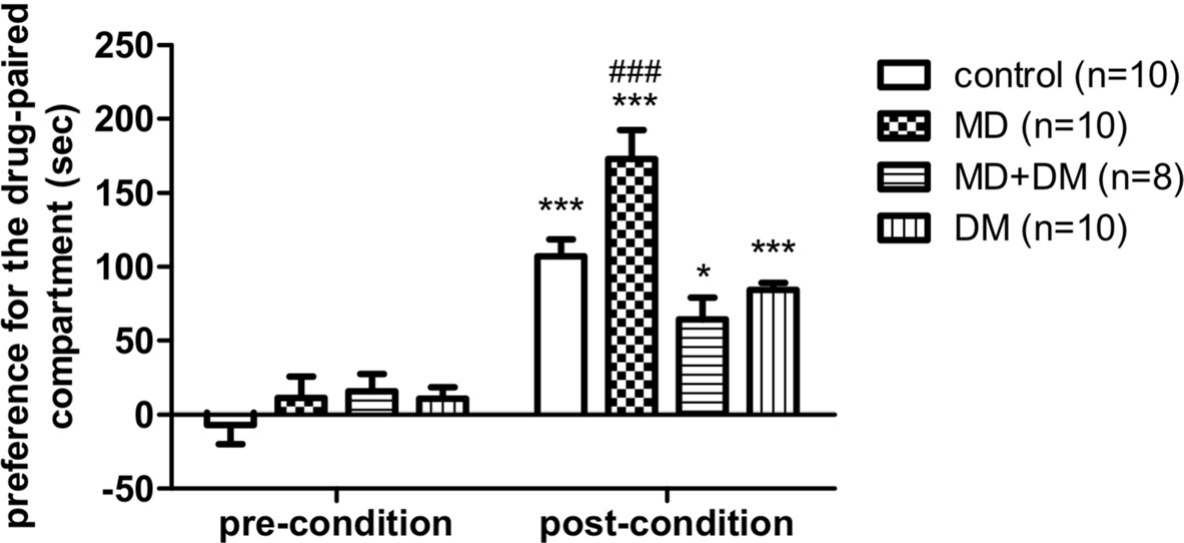
**Postnatal methadone-induced rewarding effects on prenatal methadone or DM exposed offspring.** Rewarding effects induced by methadone (MD, 6 conditioning at 4 mg/kg, *s.c.*) in male adult offspring. Data are presented as mean ± S.E.M. (n ≥ 8). *P < 0.05 and ***p < 0.001 when compared to pre-conditioning data of its own group; ^###^p < 0.001 when compared to post-conditioning data of the control group.

The CPP test was used to determine the rewarding effect of methadone in the offspring. We found that when methadone (4 mg/kg, s.c.) was given once daily and conditioned for 6 days, it significantly increased the preference time for the drug-paired compartment in all groups tested (F_(3,68)_ = 6.59; p < 0.001) (post-condition vs. pre-condition, Figure 4). However, methadone-induced CPP in the prenatal methadone-exposed group was significantly higher than that of the control group. Co-administration of DM with methadone in the maternal rats significantly prevented this higher rewarding effect and the higher addiction potential of methadone in the offspring, as shown in Figure 4 (F_(3,34)_ = 11.89; p < 0.001).

### Sub-chronic treatment with methadone in offspring induced tolerance, which was not affected by prenatal co-administration of DM with methadone

According to the results shown in Figure 2, prenatal exposure of methadone decreased the pain threshold (*i.e.* increased the pain sensitivity). In this study, the methadone-induced tolerance was measured in offspring of all groups. As shown on Table 3, the ED_50_ of all the groups before the CPP experiment were similar (1.58–1.77 mg/kg). After the CPP experiment and sub-chronic treatment of methadone, the ED_50_ of all the groups increased 1.34–1.84-fold, as shown on Table 3. However, there were no significant differences among the groups regarding the ED_50_ after sub-chronic methadone administration.

**Table 3:** **Methadone-induced tolerance in male offspring**

		**ED**_**50**_ **(mg/kg)**	
**Prenatal treated**	**Before-CPP**	**After-chronic treatment**	**Degree of tolerance (after-chronic/before-CPP)**
Control	1.77 ± 0.13	2.46 ± 0.13**	1.34
Methadone	1.77 ± 0.13	3.08 ± 0.13***	1.74
Methadone + DM	1.78 ± 0.13	3.28 ± 0.18***	1.84
DM	1.58 ± 0.18	2.46 ± 0.13*	1.56

Tail-flick test and up-down method were used to determine the ED_50_. Data are presented as mean ± S.E.M. (n ≥ 6). Two-way ANOVA and Bonferroni post-tests were used to analyze the data. *P < 0.05, **p < 0.01, and ***p < 0.001, compared to the data before CPP of its own group.

### Prenatal exposure with methadone or DM did not influence the spatial memory of male offspring, as determined by the water maze test

**Figure 5:**
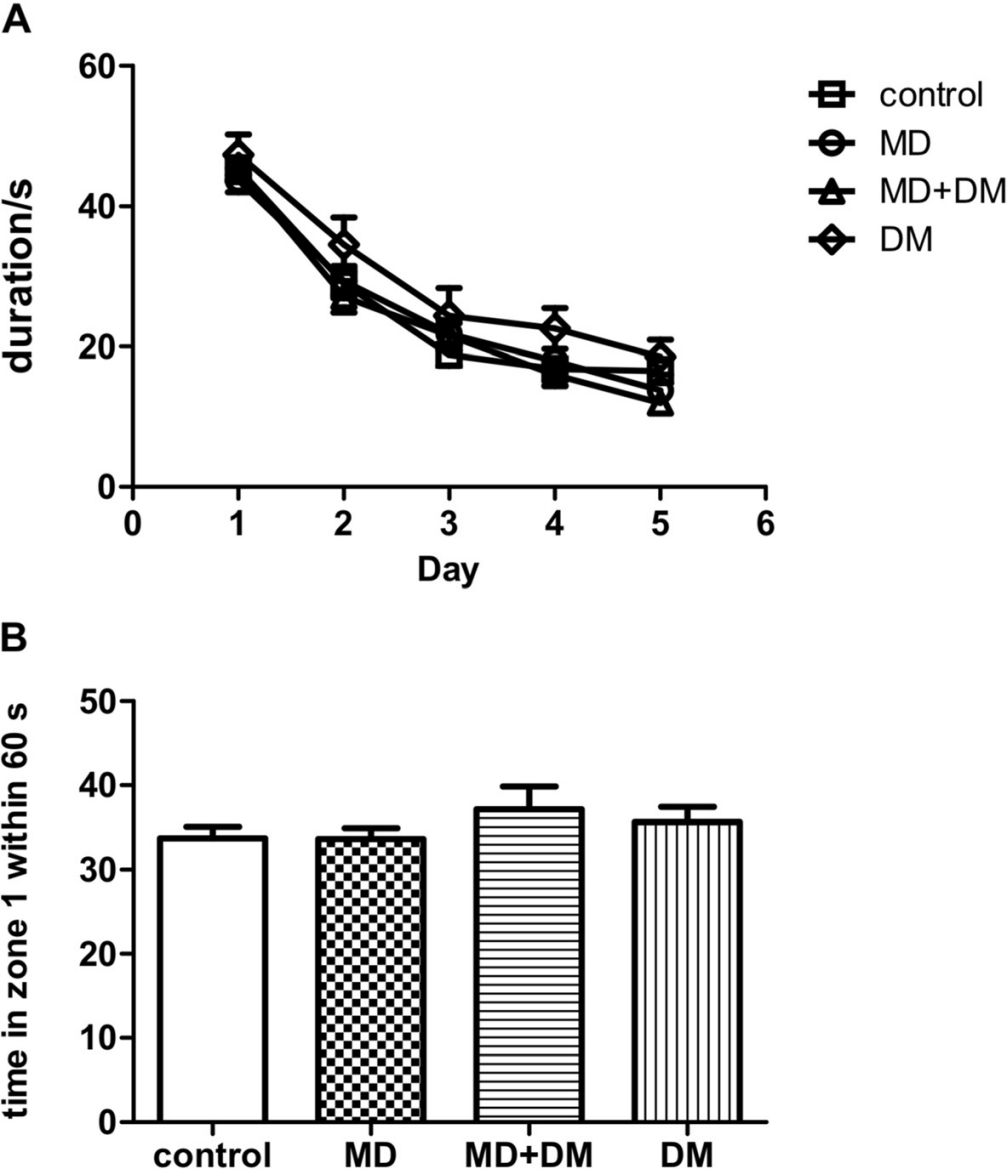
**The effects of prenatal drug exposure on spatial learning and memory of male offspring.** Prenatal exposure to methadone did not affect spatial learning and memory of male offspring determined by water maze test. **(A)** Water maze training with platform from day 1 to day 5; **(B)** water maze test without platform on day 6. Data were presented as mean ± S.E.M. (n ≥ 12).

**Figure 6:**
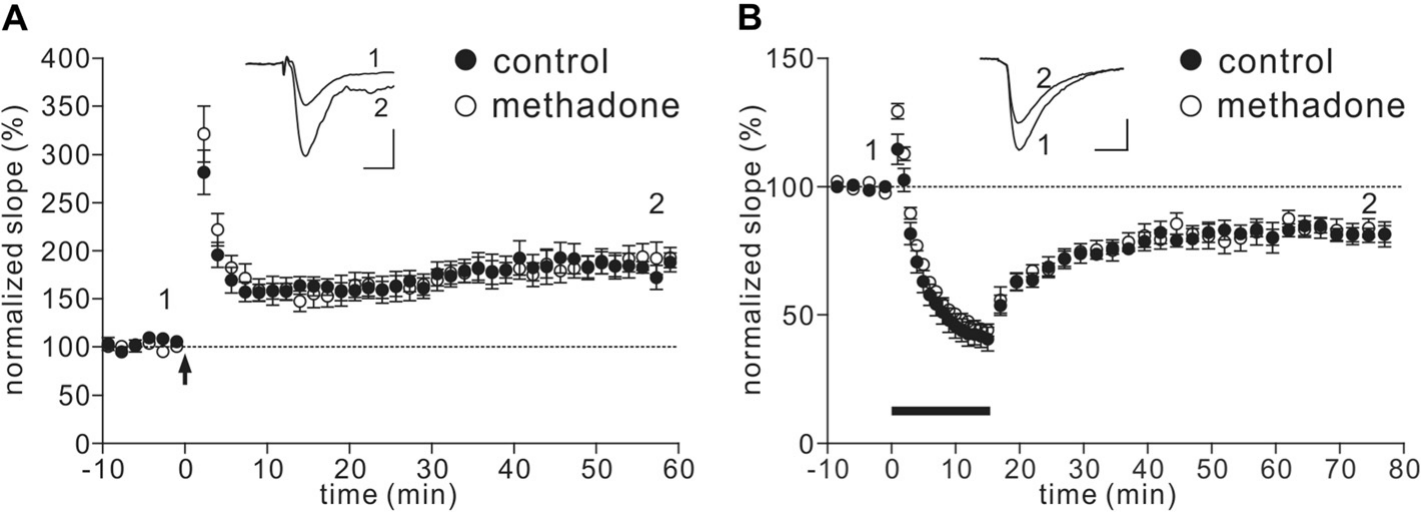
**Changes of LTP and LTD in the prenatal methadone- or DM-exposed offspring.** Prenatal methadone did not change the hippocampal LTP or LTD in p14-p21 rats. Time courses of normalized slopes of fEPSPs during **(A)** LTP and **(B)** LTD experiments are shown. A high-frequency stimulation (HFS) protocol (A) or a low-frequency stimulation (LFS) protocol **(B)** was applied at 0 min (arrow) to hippocampal slice of p14-p21 offspring born from control (black circle, n ≥ 8) or methadone (hollow circle, n ≥ 7) treated maternal rats. Representative traces of fEPSP from one control slice before HFS (1) and 55 min after HFS (2) are shown in the top middle of **(A)**, and another representative traces of fEPSP from one control before (1) and 60 min after (2) LFS are shown in the top middle of (B). Scale bar, 100 μV, 10 ms.

A water maze was utilized to test the spatial learning and memory in prenatal methadone-exposed offsprings. We found no difference in the water maze test among all groups tested (Figure 5A and B). As shown in Figure 5A, the learning rate during the five days of training was similar (F_(3,325)_ = 0.66; p = 0.9226) and there was also no significant difference for time spent in the platform quadrant (without platform) on day 6 (F_(3,65)_ = 0.89; p = 0.45) (Figure 5B) in all groups. Long-term potentiation (LTP) and long-term depression (LTD) were determined for the possible neuronal mechanisms of memory formation using hippocampal slices of prenatal methadone- or vehicle-exposed offspring (p14-p21). As shown in Figure 6, there was no significant difference between these two groups for LTP (p = 0.511) or LTD (p = 0.753).

## Discussion

The goal of this study is to evaluate the effects of co-administration of DM with methadone in the prenatal stage on offspring [21,29,31]. The doses we used are within the clinical therapeutic ranges converted to rats based on the body surface area. Seven mg/kg methadone for rats is approximately 70 mg methadone for 70 kg human; and 3 mg/kg DM for rats is about 30 mg for 70 kg human. In the present study, the findings indicate that DM treatment with methadone in the prenatal stage could prevent or suppress methadone-induced behavioral abnormality in the offspring. Furthermore, the effects of prenatal exposure to methadone to decrease the mRNA levels of MOR and NOPR in the spinal cord of offspring were also prevented by co-administration of DM with methadone in the maternal rats. Prenatal methadone did not affect learning or spatial memory in the offspring or the LTP or LTD of hippocampal slices of the prenatal methadone-exposed offspring.

The survival rate and body weights of offspring in methadone group were not different from those of the vehicle control group. Prenatal exposure to DM or methadone + DM at the doses we used did not change the survival rate or body weight of offspring (Figure 1). Furthermore, we did not observe the dams in the groups behaving abnormally during their pregnancy or breeding period. These results may rule out the possible effects of nutrition, doses of methadone, and maternal care on the behavioral changes of offspring. Therefore, the preventive effects of DM on prenatal methadone-induced behavioral or biochemical changes could be explained by its pharmacologic actions.

The changes in pain sensitivity in prenatal methadone-exposed animals might be because methadone changes the expression of opioid receptors. MOR and NOPR are expressed early in the brain and spinal cord at the embryonic stage [32,33]. These findings suggest that MOR and NOPR may be easily influenced by prenatal opioid exposure. Besides, the expressions of opioid receptors in the central nervous system may exhibit a region specific character in the intrauterine opioid-exposed animals. An early study showed that prenatal methadone administration induced sustained decreases in the expression of both delta- (δ-) and μ-opioid receptors in the hypothalamus but not in the cerebral cortex [34]. Another study by Darmani et al. [35] exhibited non-significant changes in the expression of MOR, but reducing its binding affinity to methadone in the whole brain homogenate of prenatal methadone-exposed rat offspring. Recently, Chiang et al. [36], also showed that expressions of MOR and NOPR were not different in the nucleus accumbens of prenatal methadone-exposed rats at their adulthood as compared with their control mates. In the current study, we found a significant decrease in the mRNA expression of MOR and NOPR in the spinal cord of prenatal methadone-exposed offspring at p30 and meanwhile, the pain sensitivity of offspring is increased. It has been know that the peptide of nociceptin/orphanin FQ (N/OFQ) acts on the NOPR and also serves as a pain modulator in the central nervous system [37]. N/OFQ and NOPR are highly presented in the dorsal part of the spinal cord [37,38] and the pain-modulated function of N/OFQ in spinal cord is similar with MOR endogenous ligand-endorphins [39,40]. Endorphins can act on the MOR and produce antinociceptive function in the spinal cord [40]. Early studies also showed that intrathecal administration of N/OFQ causes the antinociceptive effect in the monkey and rodents [37,38]. Both of MOR and NOPR could be influenced on expression or affinity by chronic administration of exogenous opioids [41,42]. Several studies have been shown to cross influence another type of opioid receptors when continued activation of one type of opioid receptors. For example, chronic treatment with kappa- (κ-) opioid receptor agonist, U50,448H, decreased the expression of both κ- and δ- opioid receptors in the spinal cord and striatum of rats [43]. It has also been shown that chronic morphine influenced the N/OFQ systems [42,44]. Previous studies also provided evidence that NOPR and MOR not only formed a heterodimer with each other, but also regulated the desensitization function with each other [45-47]. Formation of NOPR/MOR heterodimer selectively induced cross-desensitization of MOR and impaired the potency of DAMGO-induced adenylate cyclase inhibition and p42/p44 (MAPK) phosphorylation [46]. Furthermore, Mandyam et al. [45] found that the PKC signals may be a cross-talk mechanism for NOPR/MOR heterodimer.

On the other hand, although our results showed that the mRNA of NOPR and MOR were also reduced in the spinal cord of prenatal DM-exposed rats, the pain sensitivity did not show significant change in this group of animals. These results imply a complicated cross-talk of cellular signaling to behavioral expression and need further exploration. In a brief summary, the increase of pain sensitivity in the prenatal methadone-exposed animals may be partly due to the reduction of both NOPR- and MOR-related signals.

Besides pain regulation, prenatal opioid-exposed offspring might also change their reward sensitivity to opioids, such as offspring from morphine-addicted mother becoming more sensitive to morphine-induced reward [48,49]. In this current study, prenatal methadone-exposed offspring showed higher reward than offspring from other groups after conditioning with 4 mg/kg methadone for 6 days. This implies that the offspring from methadone-treated mothers may more easily become addicted to methadone later in their life than others.

Several studies have found that the NAS of offspring was related to the dose of methadone used in the mother [18,19,50]. A higher dose of methadone exposure in prenatal stage caused more serious NAS and increased frequency of morphine treatment in the hospital. The doses of methadone we used in the present study also induced certain natural withdrawal behaviors but not very serious (only “paws moving”). We also found that prenatal exposure to DM alone also increased natural withdrawal behaviors slightly. However, co-administration of DM with methadone in the maternal rats reduced these natural withdrawal behaviors of neonates to similar scores of the control group (Table 2).

Recently, the relationship between NMDA receptors and opioid systems has been studied. Morphine tolerance could be attenuated by resveratrol via inhibiting neuroinflammation and down-regulating the expression of NR1 and NR2B subunit of NMDA receptors [51]. Morphine-induced up-regulated state of NR3B in lymphocytes could also be reduced after subjects entered a methadone maintenance program [52]. This indicates that NMDA receptors play an important role to regulate opioid-induced effects and are involved in the opioid-addiction process in both humans and animals [53]. Since both methadone (racemic mixture) and DM have been shown to have NMDA receptor antagonist property [9,21], we think that blocking of NMDA receptors by methadone or DM may be involved in the increase of natural withdrawal behaviors of offspring. There was a human case report which showed DM caused withdrawal syndromes with over 10 folds (1800 mg/daily) of therapeutic dose [54]. However there is no evidence showed that the NAS has exhibited in prenatal-exposed infants within the therapeutic dose ranges of DM.

Compared with our previous prenatal morphine-exposed studies [28,31], methadone seems to show less adverse effects than morphine in these intrauterine exposed rats. This may be due to the NMDA receptor antagonist property of methadone. Chronic morphine administration affected the binding properties and EPSCs of NMDA receptors in the hippocampus of offspring [31,55]. It has also been shown that prenatal exposure to morphine leads to memory problems in offspring [56-58]. However, no change regarding the spatial memory or hippocampal LTP/LTD was found in prenatal methadone-exposed rats in the current study. Although suppression of NMDA receptor activities could influence the development of neurons, over-activation of the NMDA receptors through chronic activation of opioid system may also cause serious neurotoxicity in offspring or lead to other defects in them. The excess activity of NMDA receptors induced by chronic opioid (morphine or methadone) may be counterbalanced by DM or methadone itself, so that the preventative effects of DM and less-adverse effects of methadone upon the offspring could be seen. In fact, offspring exposure to DM alone in the intrauterine stage decreased the mRNA expressions of MOR and NOPR in the spinal cord, which may also be due to blocking of the normal function of NMDA receptors.

## Conclusion

In summary, this study showed that co-administration of DM with methadone in the maternal rats significantly decreased the effects of prenatal methadone on pain sensitivity, natural withdrawal, and reward in the offspring. In conclusion, this study reveals that DM may have great potential to be used in combination with methadone maintenance therapy in pregnant heroin abusers to reduce the side effects of methadone on the offspring.

## Additional file

Additional file 1:
**Detailed methods of ED**_**50**_ **, degree of tolerance, water maze, QPCR and electrophysiology.**

## Abbreviations

CPP: Conditioned place preference
DM: Dextromethorphan
fEPSPs: Field excitatory postsynaptic potentials
HFS: High-frequency stimulation
LFS: Low-frequency stimulation
MAPK: Mitogen-activated protein kinase
MOR: μ-opioid receptor
NAS: Neonatal abstinence syndrome
N/OFQ: Nociceptin/orphanin FQ
NMDA: N-methyl-D-aspartate
NOPR: Nociceptin receptor
qPCR: Quantitative real-time PCR
