# Supplementary material for: Beneficial effects of co-treatment with dextromethorphan on prenatally methadone-exposed offspring
Source: J Biomed Sci. 2015 Mar 20;22(1):19. doi: 10.1186/s12929-015-0126-2 (PMC4376496; doi:10.1186/s12929-015-0126-2)
Supplement: Additional file 1: — Detailed methods of ED 50 , degree of tolerance, water maze, QPCR and electrophysiology. [file 12929_2015_126_MOESM1_ESM.docx]

Additional file 1

# Methods and Materials

## Determination of ED_50_ and degree of tolerance after sub-chronic treatment of methadone

The ED_50_ value was used as an index of the antinociceptive effect of the drug. It was determined by the up-down method described by Dixon [1]. A series of test levels was chosen with equal spacing between each log dose of the drug. Then a series of trials (n ≥ 6) was performed in which the drug dose was reduced when tail-flick response indicated an antinociceptive effect and increased when tail-flick response indicated no analgesic effect. Each rat underwent one trial. The tail-flick latency was recorded once or twice 10–30 min after *s.c.* drug administration. The antinociceptive effect was expressed as a percentage of the maximal possible effect (%MPE) according to the following equation: %MPE = [(test latency) − (baseline latency) / (10-baseline latency)] × 100. The animal responses were defined as significant antinociception when %MPE was 50% or greater. The ED_50_ of each group was determined 3 days before the conditioned place preference (CPP) experiment (*i.e.*, day 61) and on the day after sub-chronic treatment of methadone (4 mg/kg, *s.c.*, b.i.d. for 6 days) (*i.e.*, day 79). The degree of tolerance was defined as the ratio of ED_50_ after sub-chronic treatment (day 79) and ED_50_ before CPP experiment and sub-chronic treatment (day 61).

## Determination of the rewarding effect of methadone by conditioned place preference (CPP) test

The drug-induced rewarding effect has been used to evaluate the addictive nature of drug(s) in animals. In this study, we used the CPP test to evaluate methadone-induced rewarding effect in the adult male offspring (p64–p71). The CPP test apparatus, made from an acrylic plastic box (70×25×25 cm), was divided into three compartments. Two identically sized compartments (30×25×25 cm) were constructed at both sides, separated by a narrower compartment (10×25×25 cm). The compartments were connected by guillotine doors (10×10 cm) in the central unit. One of the large compartments was covered by mosaic-type paper (2.5×2.5 cm black and white checkered squares); the other large compartment was covered by white paper. To give more visual cues, a blue light bulb and a red light bulb were hung separately above the two large compartments. For CPP conditioning, the rats were given saline in the morning and drug (methadone, 4 mg/kg, *s.c.*) in the afternoon for six days. A distinctive environment was paired repeatedly with administration of saline, and a different environment was associated with drug injection. The animals were kept for 40 min in the corresponding compartment with the guillotine doors closed. CPP tests were performed on the day before conditioning and the day after conditioning for 6 days. Place preference was determined by placing the rat in the central compartment of the apparatus with the guillotine doors open for 15 min. The measurement of the drug-rewarding effect was determined by the increase in time spent in the compartment previously paired with drug injection over the time spent in the saline-paired compartment.

## Water maze test

A water maze test was used to measure the spatial learning and memory in the young offspring (p33–38). The water maze consisted of a circular, 180 cm diameter and 60 cm deep, black-painted plastic pool filled with water (21–23 ºC) to a depth of 40 cm. The pool was shielded from the experimenter by room dividers. Spatial cues were located on the walls. The pool was viewed from above by a WV-BP330 tracking camera (1/3" CCD Digital Signal Processing). Several zones were defined within the software, and the total pool was divided into 4 quadrants (NE, SE, SW, and NW), which were designated as zones 1–4, respectively. The platform zone was set as zone 5. A calibration line (ticked line across center) was added to allow the software to convert pixel distances into physical distances. A circular transparent platform (12 cm diameter) was placed in the pool (NE quadrant, *i.e.*, zone 1) 2 cm below the surface of the water and maintained in a constant position during training. Rats received 5 days of training with two trials per day. For one trial, the animals were trained to find the platform starting from four different points distributed in the four quadrants of the pool. The animal was put into the pool and facing the wall at the beginning of each trial. The time for the animal to find the platform was recorded. During training, the maximal duration for each trial was 60 sec, after which an animal that failed to find the platform would be put on the platform for 10 sec to allow the animal to look around. On day 6, the platform was removed and the time the animal spent in zone 1 (NE quadrant, which is where the platform was located previously) was determined. All parameters were automatically recorded and analyzed by video tracking software (Etho vision, Noldus, Leesburg, VA, USA).

## Electrophysiology

Transverse 350-μm-thick hippocampal slices were prepared from p14-p21 male offspring using a commercial vibratome (DTK-1000, Dosaka, Kyoto, Japan). Animals were sacrificed by rapid decapitation, and the brain slices were cut in ice-cold artificial cerebrospinal fluid (ACSF) consisting of (in mM) 125 NaCl, 25 NaHCO_3_, 1.25 NaH_2_PO_4_, 2.5 KCl, 25 glucose, 2 CaCl_2_, 1 MgCl_2_, and 0.003 glycine. They were then incubated in a holding chamber filled with the oxygenated (95% O_2_/5% CO_2_) ACSF at 34 °C for 25 min, and then recovered at room temperature for 1 h. During the experiment, a slice was transferred to a submerged recording chamber and was constantly perfused with oxygenated ACSF at a rate of 2 ml/min at room temperature. Tungsten bipolar electrodes (FHC, Bowdoin, ME, USA) were placed in the stratum radiatum of the hippocampal CA1 region to stimulate the Schaffer collateral pathway directly. Recording electrodes with resistance of 2–6 MΩ were pulled from borosilicate glass tubings (outer diameter 1.5 mm, inner diameter 1.12 mm; FHC, Bowdoin, ME, USA) using a Flaming/Brown micropipette puller P-97 (Sutter, Novato, CA, USA). For extracellular field potential recording, a glass pipette filled with oxygenated ACSF was placed in the stratum radiatum of the CA1 area. Field excitatory postsynaptic potentials (fEPSPs) were low-pass filtered at 1 KHz using a MultiClamp 700B amplifier (Molecular Devices, Sunnyvale, CA, USA), digitized at a sampling rate of 5 KHz, and analyzed offline using a commercial software pClamp 10.2 (Molecular Devices).

fEPSPs were recorded in the presence of GABA_A_ receptor blocker, SR95531 (1 μM) and were elicited at a frequency of 0.033 Hz (every 30 sec) by constant voltage pulses generated by a square pulse stimulator S48K (Grass Technologies, West Warwick, RI, USA) connected to a stimulation isolation unit SIU5 (Grass Technologies). Stimulation intensity was set at 50% of maximal intensity that elicited population spike in CA1 striatum radiatum area. After 30 min of stable baseline recording, a high-frequency stimulation (HFS, consisting of two trains of 100 Hz bursts, 1 sec in duration and with a 1 min span between the two trains) or a low-frequency stimulation (LFS, 1 Hz for 15 min) was applied to Schaffer collaterals to induce long-term potentiation (LTP) or long-term depression (LTD) of EPSPs, respectively. A representative fEPSP trace was the average of 20 single fEPSP traces. The average of the last 5 min of every experiment was calculated and was used for analysis by Student t test.

## RNA extraction

Total RNA was extracted from the spinal cord using TRIzol reagent and following the protocol from Invitrogen (Carlsbad, CA, USA). Frozen tissue samples were homogenized with MagNA Lyser Green Beads (Roche applied science, Perzberg, Upper Bavaria, Germany) in TRIzol. Homogenized samples were transferred to fresh Eppendorf tube, diluted by chloroform, and then centrifuged at 12000×g for 15 min at 4 °C. Aqueous phase containing RNA was transferred to the fresh Eppendorf tube, and RNA was precipitated with isopropyl alcohol (1 ml per 1 ml of TRIzol), incubated at room temperature for 10 min, and centrifuged at 12000×g for 10 min at 4 °C. Supernatant was removed, RNA was washed with 75% ethanol twice, and the pellet was dissolved in RNase-free water and stored at −80 °C. Concentration of RNA was determined using the NanoDrop 2000 (ThermoFisher Scientific, Waltham, MA, USA).

## Quantitative RT-PCR

First strand cDNA was synthesized from 1 μg of the total RNA with iScript^TM^ cDNA Synthesis Kit (Bio-Rad Laboratories, Hercules, CA, USA). The qPCR was run on iQ^TM^5 multicolor Real-Time PCR Detect System (Bio-Rad). Each reaction contained 3 μl (1:10 dilution) sample of the cDNA product mixed with SsoFast^TM^ EvaGreen Supermix (Bio-Rad) up to total volume of 20 μl. Primer sequences for NOP receptor (*Oprl1*) (designed by Beacon Designer 7.6 [PREMIER Biosoft , Palo Alto, CA, USA] with NCBI reference sequence NM_031569.3), μ-opioid receptor (*Oprm1*) [2] and glyceraldehyde-3-phosphate dehydrogenase (*Gapdh*) (housekeeping gene) [3] are presented in Table 1. A three-step experimental run protocol was used: (i) denaturation (1 sec at 98 °C); (ii) amplification and quantification program repeated 40 times (1 sec at 98 °C; 10 sec at 57 °C); (iii) melting curve program (70–93 °C with a heating rate of 0.5 °C per 10 sec and continuous fluorescence measurement). All samples were run in duplicate. For each sample (control and experimental groups), the Ct (threshold) value was detected. The fidelity of the product was assessed by checking the melting curve. Results were analyzed using the obtained Ct values for all tissue samples. For example, the Ct value for *Oprl1* in the control group was subtracted from that of *Gapdh* to obtain the ΔCt value. The same subtraction was done in all treated groups to obtain the ΔCt. To compare the changes in expression levels of *Oprl1* (or others) between control and experimental tissues, these two ΔCt values were subtracted to obtain the ΔΔCt. The fold change was measured as 2^−ΔΔCt^.

# References

1. Dixon WJ: **Staircase bioassay: the up-and-down method.** *Neuroscience and biobehavioral reviews* 1991, **15:**47-50.

2. Barnes MJ, Holmes G, Primeaux SD, York DA, Bray GA: **Increased expression of mu opioid receptors in animals susceptible to diet-induced obesity.** *Peptides* 2006, **27:**3292-3298.

3. Fu Y, Xie C, Yan M, Li Q, Joh JW, Lu C, Mohan C: **The lipopolysaccharide-triggered mesangial transcriptome: Evaluating the role of interferon regulatory factor-1.** *Kidney international* 2005, **67:**1350-1361.
